# Practical Observations on Injuries of the Head

**Published:** 1841-10-01

**Authors:** 


### PRACTICAL OBSERVATIONS ON INJURIES OF THE HEAD. By *William Sharp*, F.R.S. Senior Surgeon to the Bradford Infirmary, &c. 8vo. London: John Churchill, 1841.

MR. SHARP treats the inexhaustible subject of Injuries of the Head in the following order:

Concussion of the Brain;
- Its Causes,
- Its Nature,
- Its Symptoms, and
- The Treatment.

Compression of the Brain;
- From Extravasation,

From Fracture and Depression,
From Suppuration.
The Operation of Trepanning.

Laceration of the Membranes, and
Wounds of the Brain.

CONCUSSION.

The causes and the nature of concussion first occupy him. He remarks how very slight degrees of violence will, in some instances, give rise to it. He cites two cases, one from Hippocrates, the other his own.

*Case* 1.—A young female, aged twenty, was playing with another female, a friend of hers, who struck her with the open hand on the forehead; she felt giddy and soon after became feverish, and complained of headache; on the seventh day, a large quantity of matter was discharged from the right ear, which gave temporary relief; the fever, however, returned, she became comatose, lost her speech, the right side of the face was contracted, she breathed with difficulty, convulsions succeeded, and death took place on the ninth day.

*Case* 2.—A boy, the son of a neighbour, about five years old, fell down a few steps, he was stunned for a short time, but soon seemed well, and no further notice was taken of it; several months after, he began to complain of head-ache, he became feverish, and notwithstanding the employment of very active depletive measures, squinting, dilated pupils, and excessively violent convulsions came on, the last fit of which continued three quarters of an hour, and ceased only with life. On examination, a "ramollissement" or softening of the central portions of the brain was found to have taken place to a considerable extent.

Mr. Sharp complains that the descriptions of concussion are too much mixed up with those of other lesions of the brain. He proposes that the term *concussion* or *commotion* of the brain should be restricted to cases of the first description, namely, an affection "of one or many functions, without perceptible change of structure in the cerebral fibre," or other morbid appearance within the cranium; or in the words of Benjamin Bell, "such a derangement of the brain as obstructs its natural and usual exertions; but which does not leave such marks of its existence behind it, as to render it capable of having its real nature ascertained by dissection."

The difficulty, we apprehend, is this, that there are so many mixed cases —cases in which the effects and symptoms of concussion are combined with those of extravasation, &c.

For the purpose of accurately discriminating the symptoms of concussion, Mr. Sharp arranges the cases into three classes.

The first class:—those cases in which alarming symptoms arise immediately on the injury being received, which either soon terminate in death, or speedily pass off altogether.

The second class:—cases of concussion in which a few days will elapse after the accident, and then violent and dangerous symptoms suddenly come on.

The third class:—cases in which after an accident has occurred, only slight complaints are made for a long time, and which are generally disregarded, until it is discovered that inflammation has been going on and is probably ending in a fatal effusion of serum, in softening of the brain, or in suppuration.

Mr. Sharp gives instances of the first class of cases, which are pretty common. The *second class* have not, perhaps, received so much attention from authors as they merit. Mr. Sharp relates a case, which we shall abridge a little.

1839, August 13th. Tuesday.—Mr. ——, a very stout person, was going to —— in a gig. The horse took fright and ran away; the reins broke; and Mr. —— threw himself out of the gig behind; he fell upon his left arm, and left side and hip, which were bruised: his head had not touched the ground, nor had it been struck, or knocked against anything. On Mr. S's. seeing him shortly after the accident, he complained very little, and not at all of his head; and having ordered another carriage and horse, he proceeded to ——, where he dined heartily. He afterwards got bled to nearly two pounds, and until he fainted. He returned home in the evening, (a distance of eight miles,) appearing scarcely at all indisposed.

He attended his counting-house on Wednesday, and, on Thursday, complained of violent pain and throbbing in the head, for the first time. He was bled again to a pound and a half; purged; and cold lotions applied to the head; he remained in bed, and took no solid food. On Friday, the pain in the head continued; with fits of dizziness and of suffocating flatulence; again bled to a pound and a half, and sixteen leeches applied to the temples, and calomel and digitalis given. Relieved by the bleeding.

On Saturday morning, he was better, but in the evening the pain had returned, with twitching of the right arm, and rolling of the head: bled to eight ounces, and again relieved. Small doses of compound tincture of camphor, and tincture of colchicum, were given during the night. He improved until Tuesday, when he was extremely giddy and almost suffocated with flatulence. Mr. S. gave him draughts with spiritus ammoniæ aromaticus, and infusion of cascarilla during the day, and applied croton oil to the nape of the neck. From this time he progressively recovered.

Speaking of the third class of cases, (too frequently, by the way, neglected, mistaken, or mismanaged) Mr. Sharp offers a few practical remarks connected with the subject of inflammation of the brain.

The first is, that if a patient, after an accident, complain of head-ache, throbbing in the head, giddiness, sickness, or any other symptom which may indicate an affection of the head, the most solicitous attention ought to be given to it by the medical attendant, and every proper measure adopted, to prevent or to remedy all inflammatory action.

The second observation is, that in inflammation of the membranes, particularly of the tunica arachnoides, the *pulse* is met with in two very opposite states; in the one it is very frequent, small, and wiry, as in inflammation of a serous membrane, in the abdomen; in the other it is remarkably slow, irregular, and depressed.

The third observation is that much more *delirium*, and particularly delirium of the violent kind, attends inflammation of the membranes, than of the substance of the brain.

"My fourth and last observation on this subject is, that when *convulsions* succeed the first stage of inflammation, the case, so far as I at present recollect, is always fatal, whether more blood be abstracted or not. These convulsions, to a careful observer, differ in appearance from those which arise from loss of blood or other cause of exhaustion. It is difficult to convey an idea of the difference in words, but I may mention one circumstance, the *eyes* are affected *first,* and more violently in the fit following inflammation, than when the cause is exhaustion." 40.

Mr. Sharp admits the difficulty of specifically stating the diagnostic symptoms of concussion and compression. He offers the following as the best account that he knows of.

In concussion the *symptoms* usually make their appearance immediately after the accident, though it not unfrequently happens that a considerable interval passes over before they come on; in compression there is generally a short interval between the accident and the symptoms.

In concussion, the symptoms if not so overpowering as soon to terminate in death, either pass away, the first signs of depression being followed by corresponding excitement, and this by complete recovery; or we may have the three stages pointed out by Mr. Abernethy, first of insensibility, second of restored animation, and third of inflammatory action. In compression, the symptoms if not relieved by active depletion, and other means, become aggravated, and death ensues either very soon, or after inflammation and frequently also suppuration have supervened.

In concussion, the *pulse* is generally not so slow, labouring, and intermittent as in compression.

In concussion, *bleeding* sinks the pulse and the patient; in compression the pulse rises and relief is afforded.

In concussion, there is commonly more *insensibility,* followed by *delirium,* than in compression.

In concussion, perhaps *vomiting* is more frequent than in compression, though it often occurs in the latter.

In concussion, there is less frequently *coma, convulsions,* and *paralysis,* than in compression.

The state of the *pupil* is an uncertain sign: it is often dilated and insensible to light, and often contracted in both concussion and compression.

After some judicious observations on the treatment of concussion, Mr. Sharp winds up with a few remarks on the *prognosis.* They are these.

Slight fractures are more serious than slight cases of concussion, for the obvious reason that they are an indication of greater violence having been done to the head; but on the contrary, the more severe forms of concussion are more alarming and dangerous than extensive fractures; the brain having generally suffered more injury in the former cases than in the latter.

An attentive practitioner will always remember, that slight symptoms coming on some days or weeks after the accident, bring with them a far more serious indication of mischief, than similar symptoms occurring immediately on the injury being inflicted; and also, that a patient who has suffered from concussion of the brain, in any of its forms, must not be considered really safe, until he has, for some time, remained perfectly well, Sir A. Cooper stated in his lectures, that "no patient is safe from the effects of concussion till fourteen days after the accident," that is, I suppose, when no symptoms, or only very slight ones have arisen; but I should feel it to be my duty to watch a patient for a much longer period than this, before I could dismiss my feelings of anxiety about him.

The worst cases of concussion;—frightful fractures with depression;—laceration of the membranes and wounds of the brain itself;—and extensive extravasation in the substance or at the base of the brain, will probably, in all ages, baffle the most skilful treatment. But a good acquaintance with surgery in its present state, with sufficient judgment, attention, and activity, will generally enable us to preserve life after accidents which fall short of these severe extremes.

### COMPRESSION.

The consideration of the symptoms offers nothing to detain us. We shall only pause to notice a case. It is brought forward as an instance of what occasionally happens—the lapse of an interval of some duration between the infliction of an injury and the appearance of symptoms of compression.

*Case.*—"Richard Garside, a stout healthy man, while driving his cart late at night, was attacked by two highwaymen, who kicked him severely on the head and face. He had a fracture of the upper jaw, several bruises on the head, and a wound over the left parietal bone, the bone itself being also slightly fissured. I saw him in the morning in consultation with two other medical men; he complained of nothing but the bruises about his face. The wounds were dressed, and in addition, he was freely bled. The next day he was so well that my proposal to repeat the bleeding was over-ruled. On the third day he complained of pain in the head, and was feverish; he was then bled; and again on the fourth day. On the sixth day he was decidedly suffering from compression, and the trephine was applied on the injured part of the parietal bone:—a coagulum of blood was found, but it was under the dura mater. He died on the eighth day." 80.

*Compression of the Brain from Depressed Bone* forms a separate section. Most of the remarks are characterised by good sense, rather than by novelty, indeed it would be difficult to attain and unreasonable to expect such.

We may mention that in cases of fracture with depression and symptoms of compression, with wound or without it, Mr. Sharp strongly recommends the *repeated abstraction of blood, in moderate quantities, at short intervals,* in order, if possible, *to prevent inflammation;* in preference to waiting until that mischief has actually commenced.

"The next most important question which presents itself, in these cases, is the necessity of trepanning, or at least for some operation to elevate the depressed bone. The experience I have had, leads me to advise that the depressed bone be elevated, whenever it is in a situation to be got at, even though it be necessary to make a wound to do so, *if serious symptoms of compression are actually present.* The conversion of a simple fracture into a compound one, and the removal of any portion of the cranium, are serious proceedings; and the necessity must be apparent which will justify their adoption;—that necessity appears to me to be the symptoms of compression being present. If, indeed, *these are not urgent,* blood-letting, the application of cold, &c. should be first tried: and sometimes they will subside, and the patient will recover, without further interference. But should this not be the case, should the symptoms be urgent, or though less alarming, continue after venesection, then the surgeon is called upon to attempt something more to rescue his patient from otherwise inevitable death." 105.

*Suppuration within the Cranium* is described, of course. We quote the following, a far from unique case.

*Case.*—1838. Some time this year, a soldier in the army in Spain, received a sabre wound, on the left side of the head, above the ear, which healed. In July, 1839, he was admitted into the Bradford Infirmary, for a copious purulent discharge from the left ear; a large abscess had also formed behind the ear, which was opened. He became delirious, or rather insane; and was so evidently suffering from compression of the brain, that an attempt was made to relieve him by applying the trephine above the ear. The bone was dead, and the dura mater detached from it, but no matter was foud underneath it, though the dura mater was punctured with a couching needle. The man was, however, materially relieved for several days; he afterwards became worse and died. On examination a very large quantity, between three and four ounces, of matter was found in the brain, and a perforation through the petrous portion of the temporal bone, into the meatus externus; the matter had passed through this opening, and escaped from the ear. The opening made by the trephine, was just on the verge of the abscess, but did not open it.

Mr. Sharp presents a concise yet a complete account of the history of trephining. The see-saw of opinions on the subject is remarkable, and not altogether uninstructive. His summary of the points at present agreed on and disputed lays the whole case before the reader. It is agreed on:—

Not to trepan in cases of concussion;

Nor in cases of compression of the brain, unless there is very good reason to expect extravasated blood immediately under the bone; fracture of the internal table; or pus, indicated by the puffy tumour, or detached pericranium.

Nor in children where there is depression only, without fracture.

Nor in simple fissures or fractures with slight depression, but without alarming symptoms;

Nor even in compound fractures without depresssion or symptoms;

And on the other hand:—

To perforate the cranium when extravasated blood or matter may be fairly expected to be found underneath; and to raise the depressed bone, in all cases of depression, whether simple or compound, where there are serious symptoms of pressure.

But it still remains an unsettled point whether,

A simple fracture with extensive or deep depression, but without symptoms of pressure, and,

A compound fracture with depression, but also without symptoms, should be trepanned or not.

*For* such a proceeding we have Sir A. Cooper, and Sir B. Brodie : *against* it, Dupuytren and Lawrence ;—and the arguments used on each side of the question, by these distinguished and experienced men, are of great weight.

"I do not flatter myself," adds Mr. Sharp, "that I can decide the question, as to the general rule; though I have not lately had any difficulty in making up my mind, in any individual case. I will only observe, that my decided preference has gradually become, more and more, *for non-interference ;* that *by strict attention to the means for preventing inflammation,* I have great confidence in such cases doing well, with the fracture left undisturbed: and that, therefore, unless I found that the depressed bone could be very easily raised, or the detached fragment removed without increasing the damage done to the dura mater, *I should not venture to do it.*" 139.

### WOUNDS OF THE BRAIN.

Mr. Sharp's practice differs from that of the majority of surgeons of the present day. After remarking that Sir B. Brodie in his admirable paper in the Medico-Chirurgical Transactions, has analysed a collection of thirty-eight cases, from various authors, in order to show that "in the very great majority of cases of wounded brain, there seems to be more wisdom in resorting to negative, than to active local treatment." In several of these cases, foreign bedies, or splinters of bone, were allowed to remain, without being extracted, and the patients recovered.—Mr. Sharp adds,

"In the cases which have fallen under my care, I have hitherto pursued a different course, and the majority of them have recovered; I regret that I have not accurate notes of them all, so that I might be able to say precisely in what proportion.

The question of removing fragments of bone or of foreign bodies can only be entertained, when those fragments are so loose as to be extracted without doing further injury to the brain; the result of my own practice is in favour of their being removed; while the result of my reading is, on the contrary, in favour of their being left. There can be no doubt of the propriety of leaving, unmolested, all cases where the depressed bone, or the foreign body, a bullet for example, is so circumstanced that, to extract it, the brain must suffer still further laceration.

It will perhaps appear, prima facie, that I have pursued an opposite course of practice in these cases to that recommended in the preceding chapter; but when it is considered how different the cases are;—that in the former, the brain is only *pressed upon,* and that without its giving evidence of suffering; while in the latter it has a sharp jagged edge of bone continuously *puncturing* it;—it will not then appear surprising if a different mode of treatment be thought necessary." 154.

Mr. Sharp gives a caution in reference to these, and all other fractures of the skull, where the patient recovers with loss of some portion of the bony covering of the brain. It is highly necessary *that the patient should be provided with a metallic covering for the part which has lost its bony protection,* before the surgeon takes leave of him, and before he is allowed to go much out of doors, or to engage in any active occupation or amusement. The necessity for this precaution is illustrated by a case.

*Case.*—1828. "A boy about eight years old, received a severe blow on the left parietal bone by the fall of a coal, while employed at the bottom of a pit.

I found a large wound, a comminuted fracture of the bone, and a laceration of the dura mater; he was in a state of extreme exhaustion, and in fact, it seemed a hopeless case. I removed the splintered bone, and lightly dressed the wound, and to my great surprise and delight, the boy recovered. He got out of doors, before I was aware of it, and, boy-like, engaged in full play, with some of his companions; he was struck by a stone, on the very spot where the bone had been removed, and he died in a few days.

I mention this painful circumstance, believing that it will produce as strong an impression on the minds of other surgeons, as it has upon my own; and in the hope that we may be able so to put our patients on their guard, that a similar catastrophe may, in future, be prevented. A small plate of tin, or other metal, may be made to fit over the part without difficulty. I am at present attending a case of this kind, in which, in consequence of a blow from a stone, received nearly three years ago, several large portions of the parietal and temporal bones have exfoliated, and have been extracted, by which the dura mater is exposed to a great extent; it is now protected by a large metallic plate which is constantly worn." 156.

In Mr. Sharp's cases of wounded brain, the splintered and depressed bones have been carefully removed, in the manner previously described; the integuments replaced as much as possible; the circulation repressed by the repeated abstraction of blood; inflammation checked by every possible means; and by patient and persevering attention for some weeks, a cure has been effected.

We believe we have noticed all the more prominent parts of the work. Mr. Sharp apologises for not only its execution but its production. Mr. Sharp, however, has enjoyed many opportunities for observation, and does right to record his experience. Every man's is valuable when honestly reported, and it would be well if more of our medical books were of this unpretending character.

